# Addition of Instant Shear Heat Milled Pregelatinized Rice Flours on a Gluten‐Free Pizza

**DOI:** 10.1002/fsn3.72006

**Published:** 2026-06-04

**Authors:** Ana Ligia Vargas, Reimi Sato, Hiroko Yano, Tomonori Koda, Akihiro Nishioka, Tetsuya Araki

**Affiliations:** ^1^ Graduate School of Agricultural and Life Sciences University of Tokyo Bunkyo‐ku Tokyo Japan; ^2^ Graduate School of Organic Materials Science Yamagata University Yonezawa City Yamagata Japan

**Keywords:** amorphous, gluten‐free, pizza, pregelatinized, rice

## Abstract

A new shear heat milling machine (SHMM) was used to produce pregelatinized flours instantly without additional water and with lower energy consumption. There has been an interest in expanding the application of Japonica rice flour. Nevertheless, its properties for bakery products are less than ideal, as it lacks the gluten protein matrix. Therefore, pregelatinized (AF) and regular (BF) flours were prepared using 2 Japonica rice varieties: Hoshiyutaka (HF) and Mizuhochikara (MF). Their physicochemical properties were determined, including crystallinity, hydration capacity, and pasting properties. These flours were applied to 100% rice pizza doughs at 0%, 10%, and 20% substitution levels of the AF. The doughs were subjected to rheological tests, such as a stress sweep and frequency sweep, and both the dough and baked pizza bases were observed using a scanning electron microscope (SEM). We observed that hydration capacity is significantly increased in MAF and HAF. The AF increased the dough's viscosity in rheological tests, with 20% substitution having the most notable effect. SEM images show more isolated starch granules in 0% substitution samples, whereas 10% and 20% show them covered by other materials, which we hypothesize are free starch chains of the AF. That extra structure aids the dough's manageability during processing and even baking results. In the SEM images for the baked samples, regular 0% samples showed coalesced bubbles and cracks, while 20% showed flattened small cells that were unable to expand. Therefore, we conclude that a 10% substitution had the best inner structure, with small, round cells.

## Graphical abstract

A new method of producing pregelatinized flours (AF) was developed, using a Shear Heat Milling Machine (SHMM). SHMM diminishes or even eliminates crystallinity, achieving pregelatinization. AF was incorporated into a GF pizza dough in 0%, 10%, and 20%, where it increases the viscosity and manageability of GF doughs. Free starch chains cover the native starch granules, creating structure in the dough. A 10% substitution improves the structure and gas retention of the GF pizza bases.

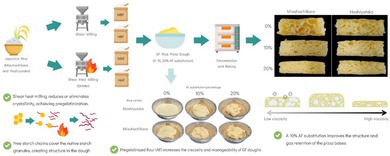

## 1 Introduction

Pizza is a traditional Italian dish originally made with a wheat base and a variety of toppings. It has become widely accepted and is consumed all around the world, leading to plentiful local adaptations and variations (Pasqualone et al. 2022).

The rise in diseases such as Celiac disease and allergies has led to a rise in dietary restrictions and a higher demand for wheat‐free products. Therefore, alternative materials such as other gluten‐free grains have quickly become a focus of study. Rice flour has been one of the most studied raw materials in gluten‐free bakery goods due to its versatility and mild flavor (Kaur et al. 2024; Park and Kim 2023).

Nevertheless, further technological problems arise from the lack of gluten itself, as the gluten matrix gives the dough its strength, stretchability, and gas retention capabilities, which are crucial in bakery products. This causes gluten‐free products to have undesirable characteristics, such as low gas retention, texture issues, including high hardness and gumminess, as well as a lower shelf‐life, which reduces their marketability (Pasqualone et al. 2022).

As reported by Šmídová and Rysová (2022), a series of solutions have been applied to gluten‐free products, such as the use of additives like hydrocolloids, emulsifiers, and protein isolates from different sources. Additives such as xanthan gum have shown promising results in improving the product's texture, but still do not allow for a full substitution of gluten on its own. Other alternatives, such as the addition of proteins, have also shown limited benefits (Park and Kim 2023).

A different approach is the use of modified starches. Chemically modified starches have drastic improvements in starch properties for food applications. Nevertheless, consumer and environmental concerns have sparked interest in research in clean‐label starch modification, such as pregelatinization (Gałkowska et al. 2023). This process involves a prior gelatinization step that breaks the original native starch granule structure into a series of amorphous starch chains; therefore, it is sometimes referred to as amorphization (Yan et al. 2024).

Up until recently, pregelatinization techniques involved heating the grains in water to allow for the gelatinization process to occur either by spray drying, drum‐drying, or extrusion (He et al. 2020; Nakorn et al. 2009; Yan et al. 2024). The obtained cooked grains or slurry were then dried and milled to obtain pregelatinized flour. This is a lengthy process that requires high water and energy consumption, resulting in a costly product.

Therefore, research aimed for an approach where the raw material didn't require rehydration and then drying. Ball milling was first studied, allowing the material to be pregelatinized after several hours of grinding. Nevertheless, this technique has high energy and time requirements to prevent overheating and the degradation of the present starch, which makes it costly and inefficient (de Oliveira Barros et al. 2023; González et al. 2018; Loubes et al. 2018).

Finally, shear heat milling was developed, in which dry grains can be converted into pregelatinized flour instantly, obtaining positive results (Katsuno et al. 2010). SHMM requires no additional water and highly reduces energy costs and production time, as drying is not required. This process can pregelatinize the starch present without much damage, allowing the resulting flour to have novel, improved properties. It has mainly been utilized to pregelatinize Haenuki rice flours (Kanke et al. 2021; Suzuki et al. 2023) as well as cellulose (Ochiai et al. 2019). Nevertheless, studies on its possible applications and utilization are still insufficient. In this study, we applied this novel raw material to pizza dough, aiming to improve its physicochemical and technological characteristics.

Furthermore, pregelatinized flour can help improve the properties of gluten‐free pizza without the use of other additives, allowing for a clean label. This is of utmost importance, as having a clean label or green label has been a challenge in the development of gluten‐free products (Šmídová and Rysová 2022). Therefore, the use of a clean‐label alternative with a short, easy, and cost‐effective production can represent a great advancement in the gluten‐free product industry.

## 2 Methodology

For this study, two rice cultivars (Mizuhochikara (MF) and Hoshiyutaka (HF)) were selected due to their physicochemical characteristics. Regular (BF) and pregelatinized (AF) rice flours were obtained using a shear and heat milling machine (SHMM) for both rice types. From now on, the four flour samples will be mentioned as MAF, MBF, HAF, and HBF for simplicity. Rice flour's physicochemical properties were measured by moisture analysis, X‐ray crystallography, hydration capacity, and viscoamylography.

A basic 100% rice flour pizza dough formulation was developed and tested with different substitutions of AF (0%, 10%, and 20%). The dough was subjected to rheological tests to determine its physical properties. After fermentation, scanning electron microscopy was performed on the dough to observe the bubble patterns that had formed.

### 2.1 Rice Flour Milling

Japonica rice was purchased whole in 5‐kg bags, depending on the variety. Domestic (Japanese) Mizuhochikara rice was obtained from Miyazaki Prefecture, and Hoshiyutaka rice from Saitama Prefecture. Regular (BF) and pregelatinized (AF) rice flours were obtained by using a shear heat milling machine (SHMM) (micro powder KGW‐G501; WEST Co. Ltd., Niigata, Japan) as described by Suzuki et al. (2023) in one 5 kg batch for each of the flour varieties. Shear heat milling allows us to pregelatinize the starch in the amorphous flour samples. For the regular rice flour samples, milling conditions consisted of a temperature of 8°C, a rotational speed of 90 rpm, a 70 μm gap between mortars, and a manually controlled feeding speed. Meanwhile, for the pregelatinized rice flour, the milling temperature was set to 120°C, with a rotational speed of 60 rpm, a 0 μm gap between mortars, and a feeding speed of 7 g/min to promote gelatinization.

### 2.2 Physicochemical Properties of the Rice Flour Samples

The moisture content of all flour samples was determined using an infrared moisture analyzer (FD‐720, Kett, Tokyo, Japan). A 3 g sample of the flour was dispersed and analyzed in triplicate.

Crystallinity was analyzed by wide‐angle X‐ray diffraction according to the technique implemented by Suzuki et al. (2023) using an X‐ray diffractometer (Ultima IV, Rigaku Co. Ltd., Tokyo, Japan). The test was conducted using a Cu‐Kα radiation source with a wavelength of 0.154 nm. Voltage was set at 40 kV and current at 40 mA. Samples were scanned from 4° to 40° at a scan rate of 10°/min. All samples were analyzed in triplicate at room temperature. The samples' crystallinity was evaluated using the peak separation method with the PeakFit v4.12 software (SeaSolve Software Inc., Framingham, USA), which interprets the results into crystalline reflection and amorphous scattering.

Hydration capacity was measured according to the AACC method 56–20.01 Hydration Capacity of Pregelatinized Cereal Products (AACC International 2010). A 2 g sample of the material was dispersed in 40 mL of water, mixed by inversion, and left to stand for 10 min. Next, the sample was centrifuged at 1000 × g for 15 min at room temperature using a Micro Centrifuge (MX‐305, Tomy Digital Biology Co., Tokyo, Japan). The supernatant was decanted. The tests were carried out in triplicate. Hydration capacity was calculated in the following way:

$$\begin{array}{c} \text{Hydration capacity}\\ \;=\frac{\left(\text{Weight of tube}+\text{sediment}\;\left(g\right)\right)-\left(\text{Weight of the tube}\;\left(g\right)\right)}{\mathrm{Dry}\;\text{weight of flour sample}\;\left(g\right)} \end{array}$$

### 2.3 Gelatinization Properties by Rapid Visco Analyzer (RVA)

The rice flour's pasting properties were determined according to the AACC Approved Method 76‐21 (AACC International 2010), with a constant speed of 160 rpm throughout the test (Vargas et al. 2025). The rheometer (MCR 302, Anton Paar, Graz, Austria) was fitted with RVA accessories (measuring cup CC26/ST‐SS, measuring cylinder ST24‐2D, and paddle stirrer ST24/2D‐2V‐2V), which record the sample's viscosity in agitation during the starch pasting process. The flour sample was first suspended in water on a 16% dry‐weight basis.

The test starts by stabilizing the sample at 50°C for 1 min. Then it is heated until 95°C at a constant rate of 5°C/min. It is maintained at that temperature for 5 min and then cooled back to 50°C at a constant rate of 5°C/min. Lastly, it is held at 50°C for 1 min. The tests were conducted in triplicate for all samples.

### 2.4 Dough Formulation

Pizza formulations were developed during preliminary testing. Ingredients used are regular rice flour, amorphous rice flour, water, egg, powdered skim milk, olive oil, salt, sugar, and dried yeast. Samples were prepared according to the following formulation (Table 1). After all the ingredients were measured, the dry ingredients were added and mixed with water, egg, and olive oil. The mixing was done manually with a spatula for 2 min until a uniform dough was achieved.

**TABLE 1:** Rice pizza dough formulations with different substitution percentages for pregelatinized (AF) flours.

Component (g)	Substitution percentage (%)		
	0%	10%	20%
Regular Rice Flour (BF)	100.0	90.0	80.0
Pregelatinized Rice Flour (AF)	0	10.0	20.0
Water	100.0	100.0	100.0
Egg	25.0	25.0	25.0
Olive oil	12.5	12.5	12.5
Milk powder	10.0	10.0	10.0
Sugar	5.0	5.0	5.0
Yeast	2.5	2.5	2.5
Salt	2.0	2.0	2.0

The dough was weighed for each pizza base, adding 88 g in aluminum molds with an 11 cm diameter base. The samples were fermented for 30 min in a fermentation chamber (SK‐15, Taisho Denki, Osaka, Japan) at 40°C. Afterward, they were baked at 180°C for 12 min in a gas oven (OZ100BOEC, Osaki Electric Co., Fukui, Japan). Samples were finally allowed to cool in their mold for 1 h before being demolded and packed in LDPE bags for further analysis.

### 2.5 Rheological Properties (Strain Sweep and Frequency Sweep)

Dough samples were prepared for rheological analysis, omitting the dry yeast to avoid interference with the results due to fermentation. Dynamic rheological properties were measured using a rheometer (MCR 302, Anton Paar, Graz, Austria) fitted with a 25 mm plate (Parallel Plate PP25) at a constant temperature of 25°C in triplicate. The dough was loaded between the two plates, allowing a 1 mm gap, and the excess was removed. After setting, a 5 min equilibration period was implemented to allow the sample to stabilize before testing.

First, a strain sweep was conducted to determine the linear viscoelastic region (LVE) and choose the correct amplitude setting in which further tests could be undertaken successfully without compromising the sample's structure. An LVE of γ = 0.08 was chosen in the amplitude test and kept constant for the frequency sweep. The frequencies were defined from 0.1 to 100 rad/s.

### 2.6 Scanning Electron Microscopy (SEM) Dough Microstructure Characterization

Small dough and baked base samples were frozen (*n* = 3 per sample) using liquid nitrogen and then freeze‐dried for 48 h in a freeze‐drier (DC‐401, Yamato Scientific Co., Tokyo, Japan) at −45°C and an internal pressure of ~10 Pa. The resulting material was pulverized and coated with gold with an ionic coater (IB‐2, Eiko Engineering Corp., Tokyo, Japan) for 3 min. Afterwards, samples were observed using a SEM (VE‐9800, KEYENCE Corp., Tokyo, Japan) with a voltage of 2 kV and a magnification of ×1000. A total of 3 fields were scanned for each sample replicate. For unbaked dough samples, images were selected to identify the best representation of the starch granules and accompanying structure after fermentation. Meanwhile, for the baked base sample, images with clear bubble morphology were selected.

### 2.7 Data Analysis

All data were analyzed using RStudio statistical software by performing either one‐way or two‐way ANOVA. Most analyzes treated the rice variety and the substitution of pregelatinized flour as independent variables. The analysis was carried out using sample means and a significance level α = 0.05. A Tukey‐HSD test was applied if statistical differences were found.

## 3 Results

### 3.1 Physicochemical Characteristics of the Different Rice Flour Samples

The samples exhibit differences that can be attributed to both their rice variety and the milling process applied. The moisture content of the AF samples is much lower than that of the BF samples (Table 2). This can be attributed to the applied shear milling technique, in which the temperature reaches 120°C. This causes the evaporation of the grain's original moisture, generating steam, a crucial step in the process that enables the gelatinization of the starch granules and the amorphization of the crystalline starch. Murakami et al. (2018) reported that rice with 12% moisture achieved complete pregelatinization, emphasizing the importance of grain moisture levels during this process. Both varieties achieve this minimum level; nevertheless, Hoshiyutaka's higher moisture content could be impactful in aiding pregelatinization, as it will be further discussed.

**TABLE 2:** Material characteristics of the regular and pregelatinized rice flour samples.

Samples	Moisture content (%)	Hydration capacity	Crystallinity (%)	Peak viscosity (Pa·s)	Trough (Pa·s)	Breakdown (Pa·s)	Final viscosity (Pa·s)	Setback (Pa·s)	Peak time (min)	Pasting temperature (°C)	Total setback (Pa·s)
MAF	5.43 ± 0.122^c^	4.13 ± 0.028^a^	1.56 ± 0.10^c^	3.27 ± 0.022^c^	0.66 ± 0.008^c^	2.62 ± 0.025^c^	1.20 ± 0.014^c^	2.07 ± 0.036^a^	5.57 ± 0.20^d^	0.00 ± 0.00^c^	0.54 ± 0.015^c^
MBF	14.68 ± 0.083^b^	2.43 ± 0.016^c^	10.90 ± 0.24^b^	6.57 ± 0.297^a^	2.22 ± 0.132^b^	4.35 ± 0.165^a^	5.05 ± 0.226^b^	1.52 ± 0.072^b^	6.73 ± 0.02^b^	59.85 ± 0.36^b^	2.83 ± 0.093^b^
HAF	4.99 ± 0.110^d^	4.00 ± 0.064^b^	0.00 ± 0.00^d^	1.06 ± 0.018^d^	0.26 ± 0.003^d^	0.80 ± 0.015^d^	0.61 ± 0.006^d^	0.46 ± 0.012^c^	5.88 ± 0.04^c^	0.00 ± 0.00^c^	0.35 ± 0.003^d^
HBF	15.06 ± 0.137^a^	2.47 ± 0.056^c^	13.34 ± 0.94^a^	6.02 ± 0.158^b^	2.60 ± 0.104^a^	3.42 ± 0.072^b^	6.26 ± 0.197^a^	−0.24 ± 0.091^d^	7.22 ± 0.03^a^	65.41 ± 0.18^a^	3.66 ± 0.094^a^
*p* value (cultivar)	n.s.	n.s.	n.s.	***	***	***	***	***	***	***	***
*p* value (milling)	***	***	***	***	n.s.	***	**	***	***	***	***
*p* value (cultivar × milling)	***	*	***	***	***	***	***	.	n.s.	***	***

*Note:* Mean values ± standard deviation shown. *N* = 3. Values in columns with different superscript letters were significantly different, as determined by the Tukey HSD test (*p* < 0.05).

Abbreviations: HAF, Pregelatinized Hoshiyutaka Flour, HBF, Regular Hoshiyutaka Flour; MAF, Pregelatinized Mizuhochikara Flour, MBF, Regular Mizuhochikara Flour.

* *p* < 0.05.

** *p* < 0.01.

*** *p* < 0.001; n.s., not significant (*p* > 0.05).

Results show a higher degree of crystallinity in BF, which is expected, as normal shear milling maintains most of the original crystallinity in the starch granules. Meanwhile, AF processed with SHMM shows a small to negligible amount of crystallinity, meaning that gelatinization did take place during the milling process (Chang et al. 2021).

**FIGURE 1:**
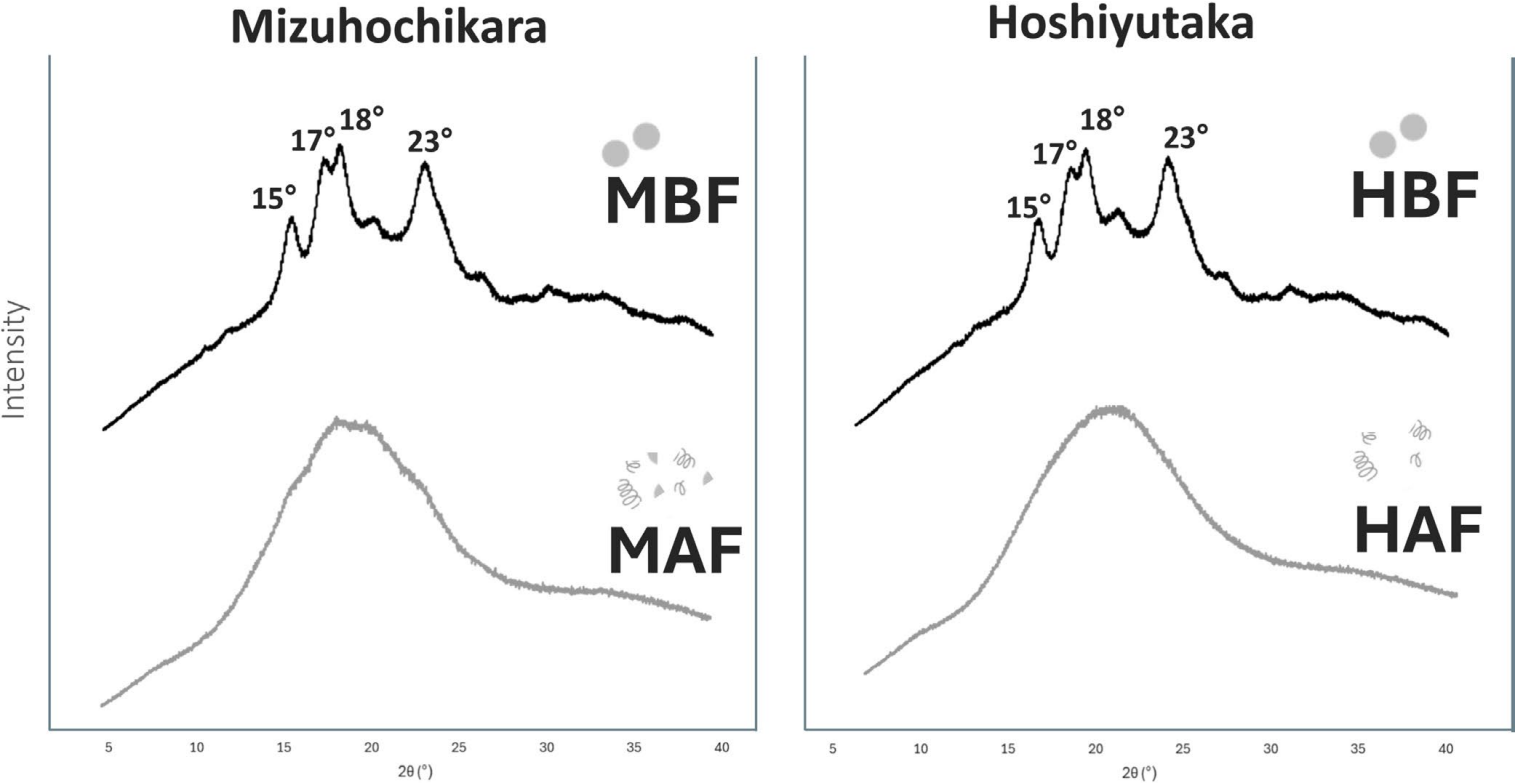
Crystallinity of the different rice flours determined by X‐ray diffraction. MAF: Pregelatinized Mizuhochikara Flour, MBF: Regular Mizuhochikara Flour, HAF: Pregelatinized Hoshiyutaka Flour, HBF: Regular Hoshiyutaka Flour.

This technique enables us to confirm that samples, such as HΑF, underwent complete gelatinization of the starch granules, resulting in the complete amorphization of the present starch, and categorize this sample as a fully pregelatinized starch. Meanwhile, MAF shows a small degree of crystallinity remaining, as confirmed by faint peaks still observable in the X‐ray diffraction patterns (Figure 1). This was probably caused by the higher content of amylopectin present in the sample, which leads to a higher crystallinity due to the side chains that form crystalline double helices (Suzuki et al. 2023). A lower initial moisture also causes the sample not to be fully gelatinized, as the fast shear heat milling processes tend to discharge the sample before complete gelatinization. The remanent crystallinity causes MAF samples to have a higher gelatinization temperature, as will be discussed in section 3.2. This higher temperature was caused by the sample, which was almost but not completely gelatinized during the milling process.

The hydration capacity of AF (MAF and HAF) is significantly higher than that of BF (MBF and HBF), with AF almost doubling due to the pregelatinization treatment. This can be explained by the higher associative forces found in granules, which don't allow the starch to bind to water easily at room temperature (Nakorn et al. 2009). A lower initial moisture and crystallinity percentage indicates more unwound and free starch, which can absorb water quickly. Therefore, it is confirmed that moisture content and crystallinity percentage inversely correlate with the sample's hydration capacity (González et al. 2018). This level of increase in the hydration capacity is similar to boiling and drying, and comparable to or even higher than steaming and extrusion, depending on the processing conditions (Matia‐Merino et al. 2019; Sun et al. 2018). This makes AF produced by SHMM a great raw material for further testing.

### 3.2 Gelatinization Properties by Rapid Visco Analyzer (RVA)

**FIGURE 2:**
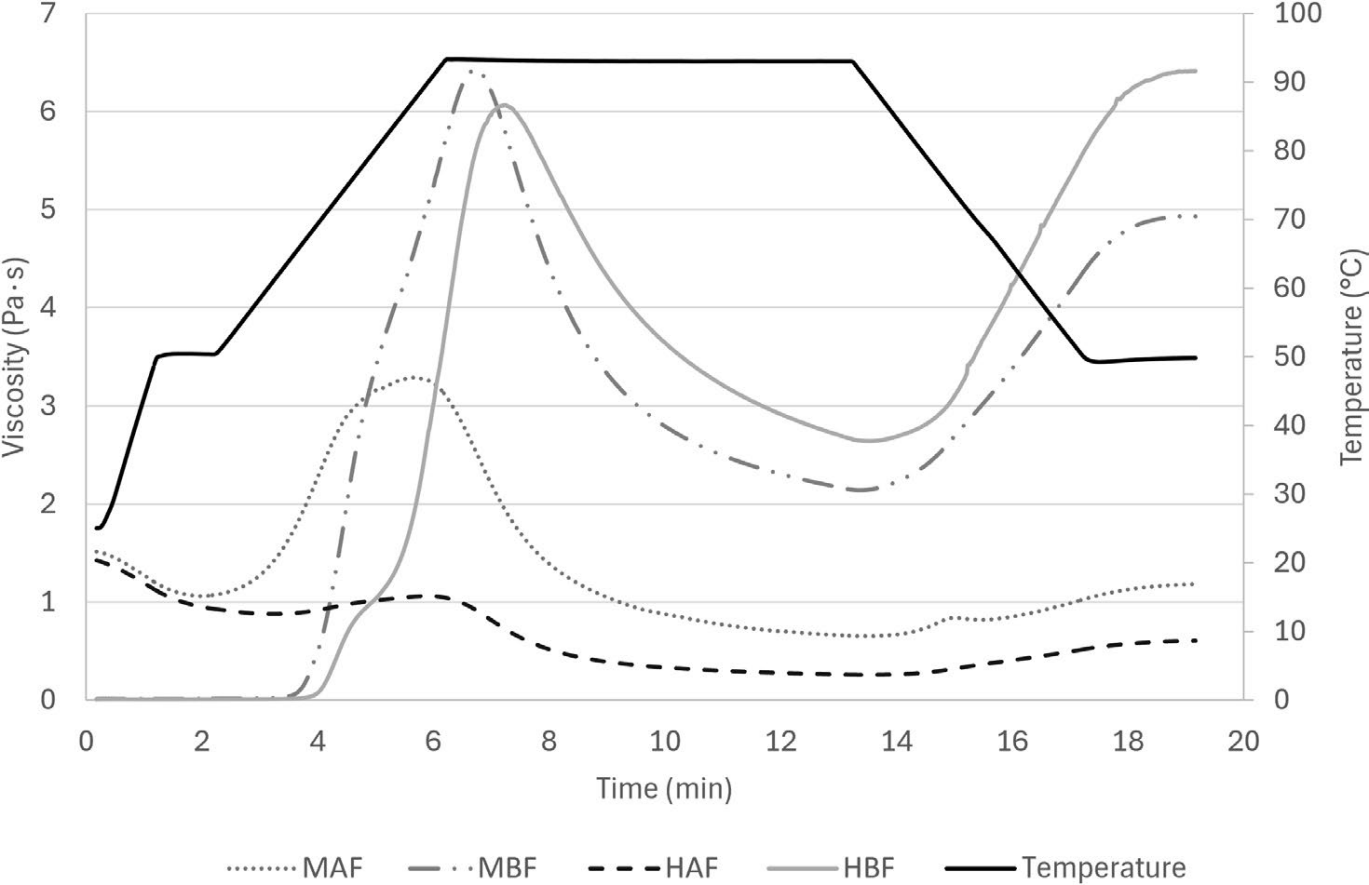
Pasting properties of the regular and pregelatinized flours determined with a Rapid Visco Analyzer (RVA). MAF: Pregelatinized Mizuhochikara Flour, MBF: Regular Mizuhochikara Flour, HAF: Pregelatinized Hoshiyutaka Flour, HBF: Regular Hoshiyutaka Flour.

RVA results show a vastly different behavior between pregelatinized and regular flours (Figure 2). Regular flour samples (both MBF and HBF) exhibit the expected behavior, characterized by a high peak viscosity at first, followed by a downturn known as breakdown, and a final rise due to setback as the sample cools and the starch chains form a gel. A higher amylopectin content in the starch composition can explain a higher peak viscosity in the case of MBF when compared to HBF (Nakamura et al. 2021).

After gelatinization, starch gels are in a very thermodynamically unstable state; therefore, as they reorganize, their final viscosity increases. Several factors contribute to the final increase in viscosity, commonly referred to as total setback. It can be observed that HBF displays a more significant total setback behavior when compared to MBF, caused by a higher amylose content, which is more prone to reorganizing in a crystalline structure due to its linear molecular structure (Chang et al. 2021).

In the case of pregelatinized flours, amorphization during milling greatly affects gelation properties. Since HAF was completely amorphized previously, the viscosity remains remarkably constant during the test, with only a slight change in the final viscosity compared to the initial one, likely due to damage to the starch structure caused by the heating and agitation performed in the amylograph test. This behavior has been reported for pregelatinized flours obtained by different techniques, such as ultrasonic‐assisted pregelatinization (Oh et al. 2024) and ball milling (González et al. 2018).

Meanwhile, MAF shows an intermediate behavior between HAF and the MBF and HBF. Due to structural damage, an intermediate peak viscosity is observed, accompanied by a reduction in viscosity similar to that seen in HAF. The intermediate peak viscosity observed can be attributed to the gelatinization of the remaining crystalline starch (Gulzar et al. 2021). Nevertheless, as the crystallinity is much lower compared to the regular samples, both HAF and MAF display the lowest and second‐lowest total setback behavior, which is considered a positive quality. Pasqualone et al. (2022) described that the substitution of some of the regular flour with an alternative flour that possesses a lower total setback is beneficial in a GF pizza formulation, as it tends to achieve products with a longer shelf life, as it prevents retrogradation and staling.

### 3.3 Rheological Properties

**FIGURE 3:**
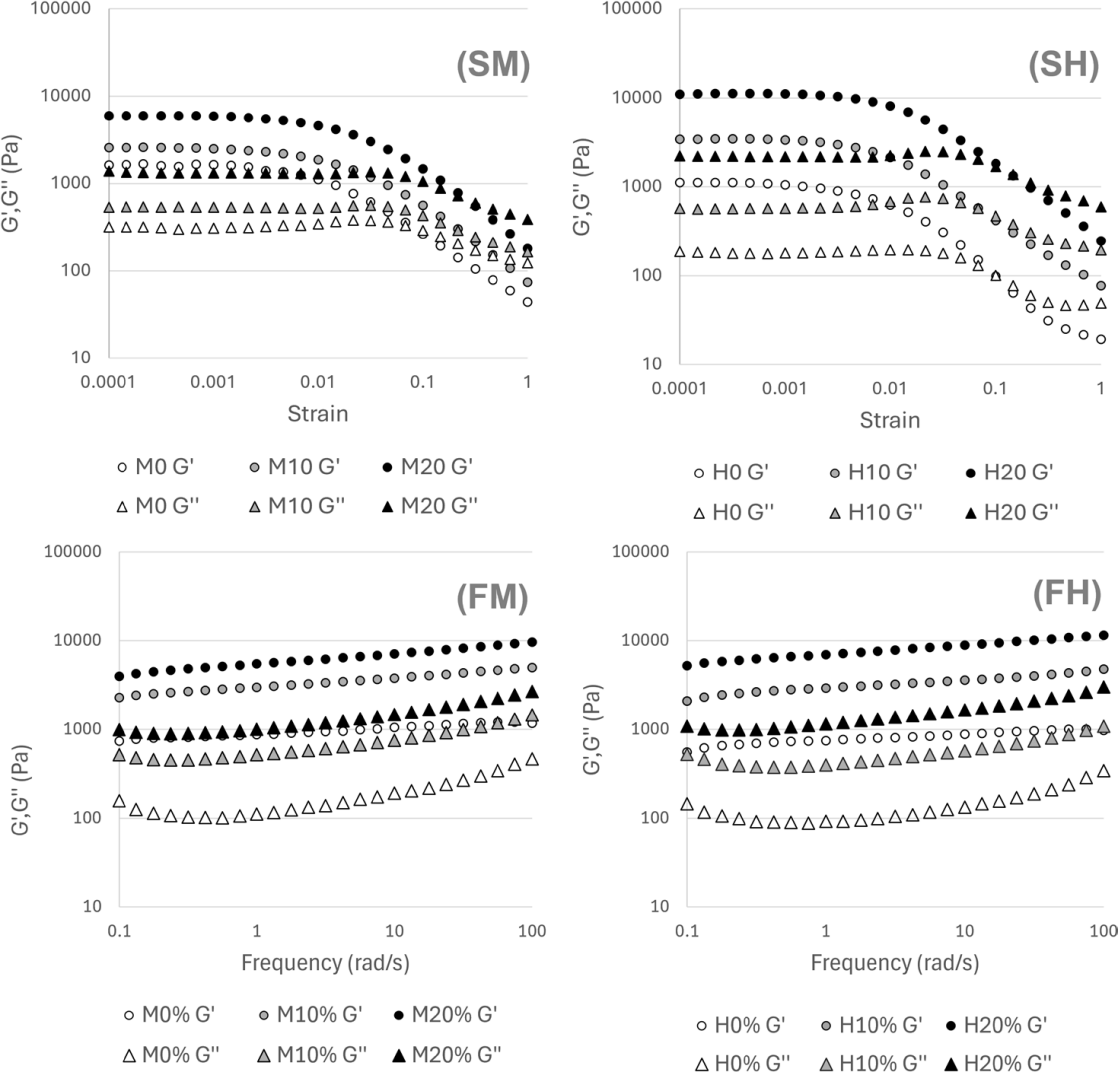
Rheological strain sweep (S) and frequency sweep (F) results for rice pizza base doughs formulated with different substitutions of AF from the following varieties: (M) Mizuhochikara, (H) Hoshiyutaka (Storage and loss modulus represented as: G′ ●, G″ ▲).

A dough's rheological properties are crucial in the development of gluten‐free products that resemble their wheat counterparts (Yazar and Demirkesen 2023). With the addition of AF, the resulting dough exhibits a visible increase in viscosity with 0% AF < 10% AF < 20% AF throughout all the tests conducted (Figure 3—FM, FH, SM, and SH). This is a key functional property of AF, as it increases the dough's manageability for manufacturing. This increase in viscosity upon addition of AF was later observed in both the strain‐sweep and frequency‐sweep results. This is shown as an increase in the G′ and G″ for the substituted samples in both tests. Notably, there are also significant (*p* < 0.001) differences between 10% AF and 20% AF substitution levels when an ANOVA was applied to four points throughout the frequency sweep analysis at frequencies: 0.1, 1, 10, and 100 rad/s. A similar result was reported by Matsumoto et al. (2022) when pregelatinized wheat starch was applied to a regular wheat pizza dough.

#### 3.3.1 Strain Sweep and Frequency Sweep

A strain sweep is conducted to determine the sample's resistance to changes in oscillatory strain amplitude, as increasing the amplitude increases the applied shear strain. In this case, a G′ > G″ is observed at the start of the test, signifying stronger elastic behavior (Ren et al. 2020). In lower amplitude regions, the behavior is linear (Figure 3—SM and SH). As strain increases, both parameters exhibit a crossover beyond the Linear Viscoelastic Region (LVE) (Matsumoto et al. 2022). Normally, an increase of the shear strain beyond the LVE leads to a modification or destruction of the samples, as the internal physical structure is damaged. In the tests performed, a breakdown of the dough's structure can be observed for all samples at an amplitude of 0.01% where a reduction in both the storage modulus (G′) and the loss modulus (G″) occurs. Therefore, a (LVE) of γ = 0.08 is selected for all further tests.

An increase in both G′ and G″ can be observed when the pregelatinized flour substitution percentage is increased. This is caused by the free starch chains that can improve the dough and foam structure, at room temperature or during fermentation (Dang et al. 2025). This also allows the dough to have a higher viscosity and better manageability.

A frequency sweep test was subsequently applied to all the dough samples. Figure 3 (FM and FH) shows the behavior of the storage modulus (G′) and the loss modulus (G″) when the oscillation frequency gradually increases. All the dough samples exhibit an increase in G′ and G″ with the increased frequency, implying that the viscoelasticity of the dough is frequency dependent. This can be due to the pregelatinized starch promoting more intermolecular cross‐linking and therefore strengthening the gel network (Li et al. 2020; Yan et al. 2024).

The biggest effect of the addition of the AF is the increase in viscosity, even from a low shear standpoint. It is worth noting that the y‐axis in such graphs corresponds to a logarithmic scale; therefore, the increase in the viscosity is quite pronounced. This effect has been observed in previous studies. It is hypothesized that the free starch chains that are released in a prior gelatinization stage generate a better structure in low‐protein doughs such as rice; therefore, increasing both G′ and G″ (de Oliveira Barros et al. 2023; Yan et al. 2024).

#### 3.3.2 Scanning Electron Microscopy (SEM) Dough and Baked Bases' Microstructure Characterization

**FIGURE 4:**
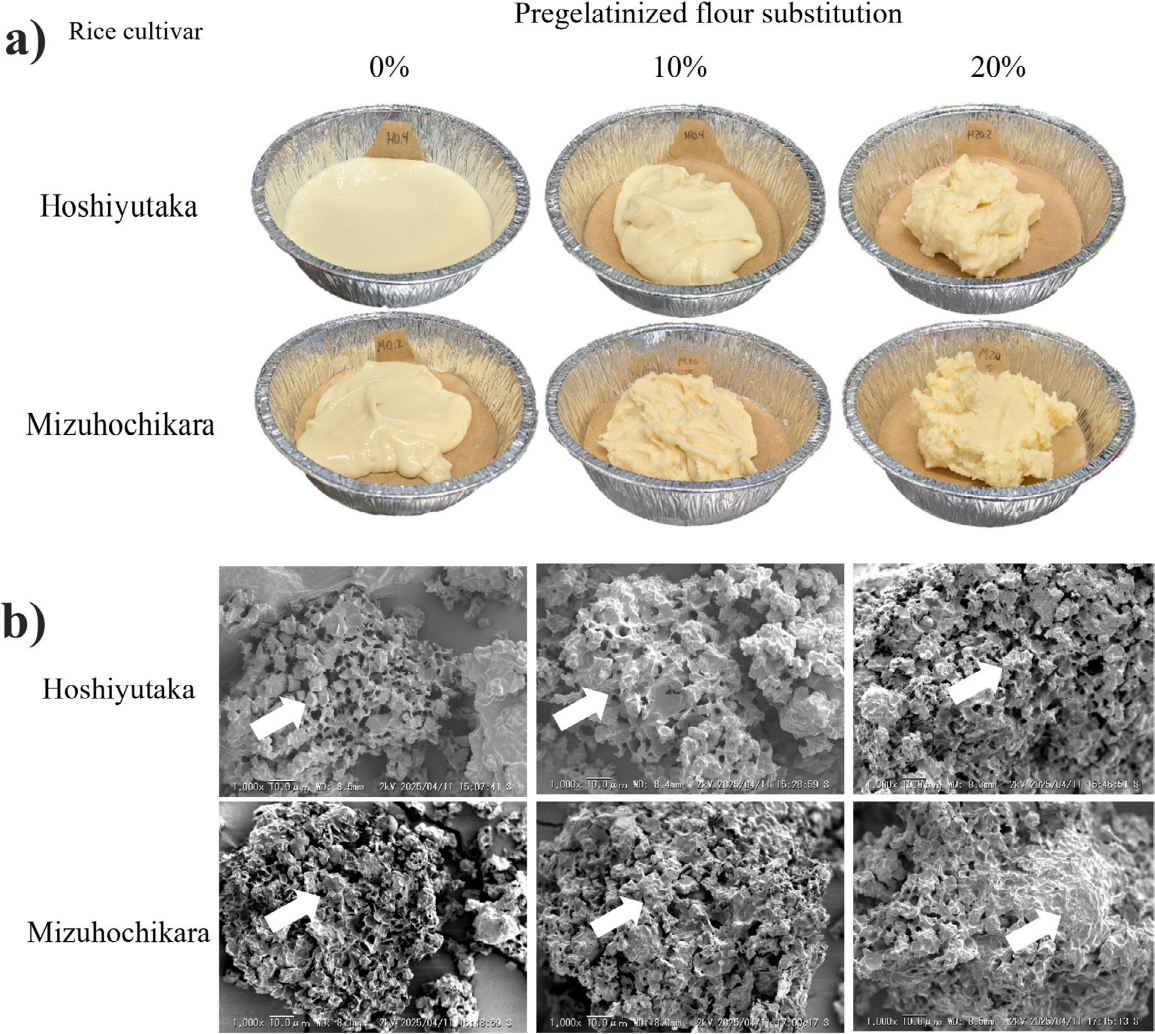
Properties of the gluten‐free rice pizza dough with different pregelatinized flour substitution percentages: (a) visual viscosity variations, (b) shape and bubble structure observed by SEM.

When observing the effects of AF on the pizza dough, scanning electron microscope images reveal that the native starch grains become progressively covered by other substances as the substitution percentages of pregelatinized flour increase (Figure 4). We hypothesize that these substances consist of a mixture of the used ingredients, heightened by the pregelatinized free starch chains available in the dough.

During previous studies, it was confirmed that AF obtained by shear‐heat milling lost the granular structure observed in regular samples (Kanke et al. 2021). An amorphous structure correlates with a high amount of free starch chains, which can easily absorb water and act as binders within the dough. The addition of these elements to the gluten‐free dough increased its viscosity, as reflected in both rheological tests. According to the behavior described by Ding et al. (2021), the rheological changes observed in the dough provide a better structure for CO_2_ retention before baking, thereby improving the baking process and the final texture of the baked goods.

**FIGURE 5:**
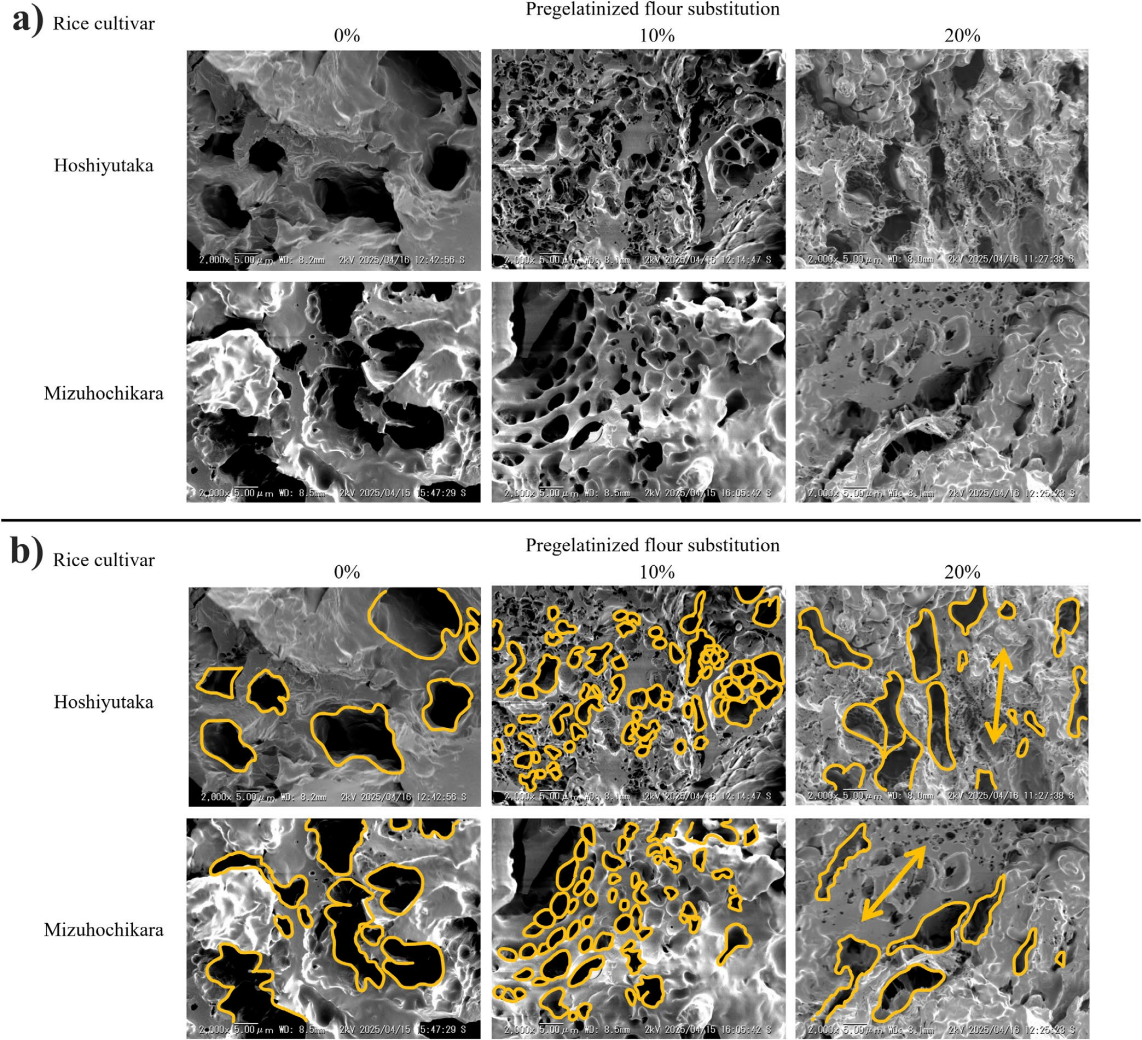
Baked gluten‐free pizza base microstructure and bubble structure observed by SEM: (a) original images, (b) highlighted images.

This effect was then confirmed when analyzing the baked pizza bases with the same methodology using SEM imaging (Figure 5). The samples with only regular rice flours present big cavern‐like structures, where several of the internal bubbles joined together forming tunnels where the CO_2_ could escape (Manik and Nur 2021). This correlates with cracks and a low volume of the pizza bases, due to the lack of structure that can retain such gas. This gas tends to escape, leaving behind an unstable structure. These structures are correlated with a lower elasticity of the sample, which can be observed with the lower values of G′ exhibited by the 0% AF samples (Yan et al. 2024).

As for the 10% AF substitution samples, small, uniform bubbles can be observed. This is an ideal situation for a gluten‐free product, as it signifies that the structure is soundly retaining the gas while expanding uniformly during the baking process, allowing for a better texture. Finally, in 20% substitution samples, the images show wider, flatter bubbles that stretch in the same direction. Such bubbles are observed when the viscosity is too high for the CO_2_ to properly expand, which restricts the dough expansion. This same effect has been observed in gluten‐free cakes with increased viscosity due to the addition of protein (Sahagún et al. 2018). It can be inferred that this result is mainly caused by a physical effect.

**FIGURE 6:**
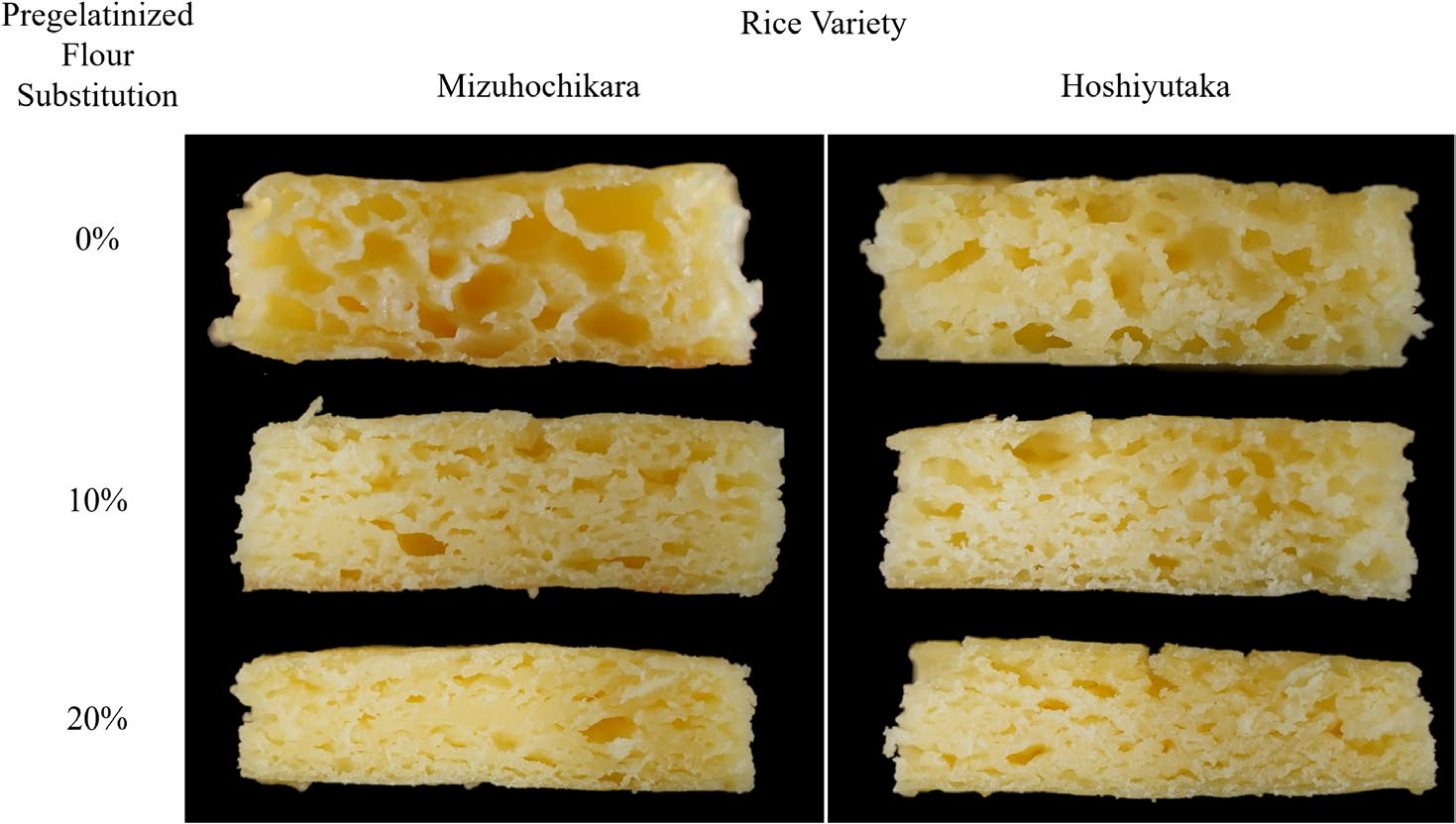
Baked gluten‐free pizza base samples' internal structure with the addition of pregelatinized flour.

It is also important to recognize that the patterns observed in SEM imaging also match the macroscopic structure present in the baked samples (Figure 6). A similar effect was observed in GF rice flour breads, in which the structure of the final product is largely determined by the bubble structure after fermentation (Saito et al. 2022). Therefore, considerations at a microscopic level prove quite significant when dealing with this matrix.

## 4 Conclusions

The use of SHMM greatly modifies the properties displayed by rice flour samples, causing more significant changes than using different rice varieties. An immediate decrease in crystallinity and flour moisture content increases the free starch chains available to readily interact with added water. This amorphization increases the hydration capacity of such pregelatinized flours, allowing for an instant thickening effect upon use. The thickening capabilities largely increase the 100% rice flour pizza dough's initial viscosity, as reflected in its rheological properties. This allows for easier management during processing and fermentation. In addition, AF can generate a viscoelastic structure between starch granules in the raw dough, as observed in the SEM images. This was later confirmed in the SEM images for the baked bases, where the structure formed was significantly different. A 10% substitution shows a promising level for structural improvement and CO_2_ retention in a rice gluten‐free pizza base.

## Author Contributions

**Reimi Sato:** methodology, formal analysis, conceptualization, software. **Tetsuya Araki:** supervision, resources, project administration, funding acquisition, writing – review and editing, software. **Hiroko Yano:** conceptualization, methodology, project administration, data curation, supervision, resources, writing – review and editing, validation. **Akihiro Nishioka:** writing – review and editing, project administration, supervision, resources. **Ana Ligia Vargas:** conceptualization, investigation, writing – original draft, methodology, visualization, writing – review and editing, formal analysis, data curation, validation, software. **Tomonori Koda:** project administration, supervision, resources, writing – review and editing.

## Funding

This work was supported by the Ministry of Education, Culture, Sports, Science and Technology (Grant 210424).

## Disclosure

Author Approval and Responsibility Statement: All authors have read and approved the final version of the manuscript. The corresponding author had full access to all of the data in this study and takes complete responsibility for the integrity of the data and the accuracy of the data analysis.

## Ethics Statement

This study did not require additional ethical clearance, as no human or animal participants were involved in the conducted tests. All presented results are based on experimental data from food samples, and no sensitive data were collected.

## Conflicts of Interest

The authors declare no conflicts of interest.

## Data Availability

The data that support the findings of this study are openly available in Figshare, https://doi.org/10.6084/m9.figshare.31323166.
